# Refractory Ranula Following Surgery for Tongue Cancer: A Case Report

**DOI:** 10.7759/cureus.86898

**Published:** 2025-06-28

**Authors:** Atsuya Ishiyama, Kunio Yoshizawa, Karen Gomi, Akinori Moroi, Koichiro Ueki

**Affiliations:** 1 Department of Oral and Maxillofacial Surgery, Division of Medicine, Interdisciplinary Graduate School, University of Yamanashi, Chuo-shi, JPN

**Keywords:** case report, intraoral ranula, marsupialization, ok-432, sclerotherapy, tongue cancer

## Abstract

The postoperative complications of tongue cancer include functional impairment due to tongue removal, sensory and taste disturbances due to damage to the lingual nerve, and intraoral ranula due to damage to the salivary gland duct in the lower part of the tongue. Surgical treatments, such as marsupialization, cystectomy, and sublingual adenectomy, have long been used to treat intraoral ranulae. Sclerotherapy with OK-432, which is less invasive than other treatments, has also recently been used. However, to the best of our knowledge, no case of sclerotherapy with OK-432 for ranulae resulting from postoperative complications of tongue cancer has been reported to date. In this report, we describe a case of a postoperative intraoral ranula that was treated several times with sclerotherapy using OK-432; however, the ranula recurred. Therefore, we performed marsupialization and obtained satisfactory results. A man in his 60s underwent partial tongue resection for right-sided tongue cancer (T2N0M0). The resected area was then covered with polyglycolic acid sheets and fibrin glue. Seven months after surgery, a well-defined, soft, blue-violet mass measuring 38 × 26 mm was observed at the resection site. Contrast-enhanced magnetic resonance imaging (MRI) of the head and neck revealed a cystic high-signal area suggestive of fluid collection in the right sublingual space on T2-weighted images. Local and imaging findings were consistent with those of the right intraoral ranula. OK-432 1 KE/0.2 ml was then injected into the right ranula. After the injection, a temporary resolution was observed; however, an increase in the size of the recurring ranula was noted. Therefore, three OK-432 injections were administered. However, owing to recurrent findings of the ranula in the same area, right-sided ranula marsupialization was performed under general anesthesia five months after the first OK-432 injection. The surgical field performs a tie-over. Contrast-enhanced MRI was performed three months after marsupialization to confirm the absence of recurrence or metastasis of the ranula or tongue cancer. Currently, 12 months after surgery, no recurrence or adverse events have been observed in either the tongue cancer or the ranula. Our case revealed that sclerotherapy with OK-432 is not effective for ranula, which is a postoperative complication of tongue cancer. In such cases, surgical treatments such as marsupialization may be appropriate.

## Introduction

Postoperative complications of tongue cancer include functional impairment due to tongue resection as well as sensory and taste disorders due to lingual nerve damage. Other complications include xerostomia and intraoral ranulae, which are associated with damage to the salivary gland conduit located on the lower tongue. The complications of tongue cancer have been well investigated with respect to improving functional impairments such as pronunciation and eating; however, ranula, a postoperative complication, has not been well investigated [1-3].

A ranula is a cystic swelling caused by damage to the ducts of the sublingual gland and leakage of saliva from the salivary ducts and glands. Clinically, it presents with repeated episodes of shrinkage and enlargement. Pathologically, the cyst wall consists of fibrous or granulation tissue; ranula is a pseudocyst lacking epithelial cells [1]. The ranulae are classified as sublingual or submandibular ranulae. A ranula is classified as an intraoral ranula if saliva accumulation occurs above the hyoid muscle and as a plunging ranula if saliva accumulates beyond the hyoid muscle [4]. Two types of treatment are considered for managing ranulae: surgical treatment and sclerotherapy using OK-432. OK-432 (Picibanil^®^) is a benzyl-penicillin-treated freeze-dried preparation of *Streptococcus pyogenes* (group A, type 3, Su strain), which was originally approved as an immunotherapeutic agent against cancer [5]. Ogita et al. [6] have reported the effectiveness of an intracystic injection of OK-432 for the treatment of lymphangioma in 1987; since then, sclerotherapy using OK-432 has been recommended as a standard treatment modality for managing lymphangioma [7]. Its use is also becoming increasingly common in cystic diseases of the head and neck region [8]. In addition to surgical treatments, such as marsupialization, cystectomy, and sublingual adenectomy, sclerotherapy using OK-432 has recently been used to treat ranulae because it is less invasive than other treatment methods [9,10]. However, we did not find any cases of sclerotherapy with OK-432 for ranulae arising from postoperative complications of tongue cancer.

This report describes a case of an intraoral ranula as a complication of tongue cancer surgery. Sclerotherapy with OK-432 was administered several times; however, the ranula recurred. Therefore, marsupialization was performed, and positive outcomes were achieved.

## Case presentation

**Figure 1:**
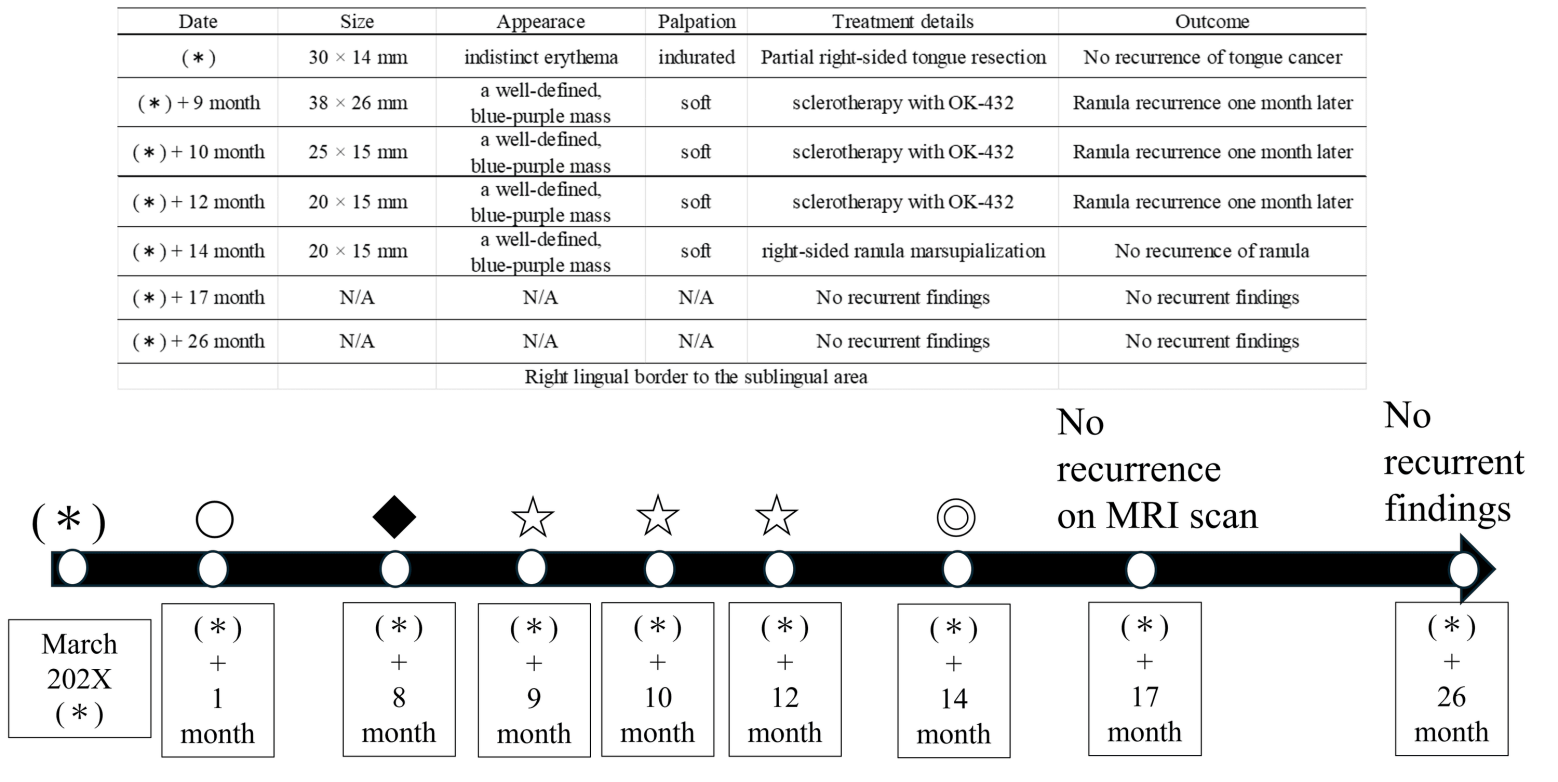
Schematic diagram and table of the treatment process. (*) Diagnosis of tongue cancer T2N0M0; 〇 partial tongue resection; ◆ diagnosis of right side intraoral ranula; ☆ OK-432 1 KE + 0.2 ml saline injection into ranula; ◎ ranula marsupialization.

A schematic diagram and table of the treatment course in this case are shown in Figure 1.

**Figure 2:**
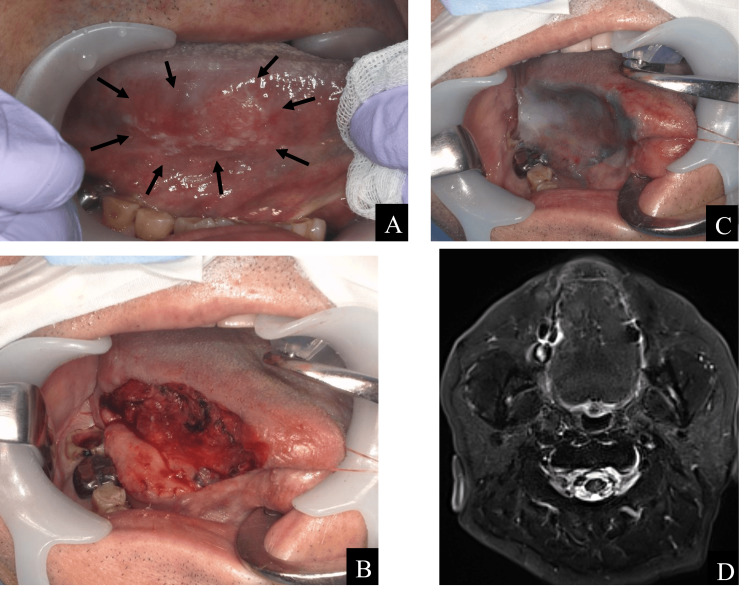
The treatment process for tongue cancer. (A) Intraoral photograph at the initial visit. The diagnosis was tongue cancer T2N0M0, with a long diameter of 21 mm and a depth of invasion of 2.6 mm. (B) Intraoperative photograph of partial resection of tongue cancer. Intraoperative rapid diagnosis of negative margins was obtained. (C) Polyglycolic acid (PGA) sheets and fibrin glue were used to cover wounds after partial tongue cancer resection. (D) Contrast-enhanced MRI, T2-weighted image, three months after tongue cancer surgery. There were no obvious recurrence findings.

In March 202X, a 62-year-old man presented with the chief complaint of pain in the right side of his tongue. His medical history included hypertension and diabetes mellitus, both of which were treated. The body size was normal, and no neck swelling was observed. Intraoral examination revealed a white lesion measuring 30 × 14 mm with indistinct erythema on the right lingual border. This area was indurated and easily hemorrhaged (Figure 2A). Because of these artifacts, it is difficult to identify the primary site of tongue cancer using computed tomography (CT) and ^18^F-fluorodeoxyglucose-positron emission tomography. No enlarged cervical lymph nodes suggestive of metastasis were observed. A biopsy was performed as tongue cancer was strongly suspected. A partial biopsy of the lesion, including the healthy tissue, was performed. Aberrant squamous cell proliferation and keratinization were observed. There was evidence of squamous cell carcinoma. He was diagnosed with T2N0M0 right-sided tongue cancer based on biopsy and imaging results.

Partial right-sided tongue resection was performed under general anesthesia one month after the initial diagnosis of tongue cancer on the right tongue margin (Figure 2B). The resection area was covered with a polyglycolic acid (PGA) sheet (NEOVEIL sheet^®^, Gunze Ltd., Osaka, Japan) and fibrin glue (BOLHEAL^®^, KM Biologics Ltd., Kumamoto, Japan) (Figure 2C). Histopathological examination of the resected specimen revealed a squamous cell carcinoma with a long diameter of 21 mm, a depth of invasion (DOI) of 2.6 mm, and an inward-directed type. The resection area was confirmed to be epithelialized one month after the operation (two months after the initial visit). The patient had introverted-type tongue cancer; therefore, TS-1 at a dosage of 120 mg/day was started as postoperative adjuvant chemotherapy (two weeks on, one week off). Contrast-enhanced magnetic resonance imaging (MRI) performed three months after tongue cancer surgery revealed no evidence of recurrence (Figure 2D).

**Figure 3:**
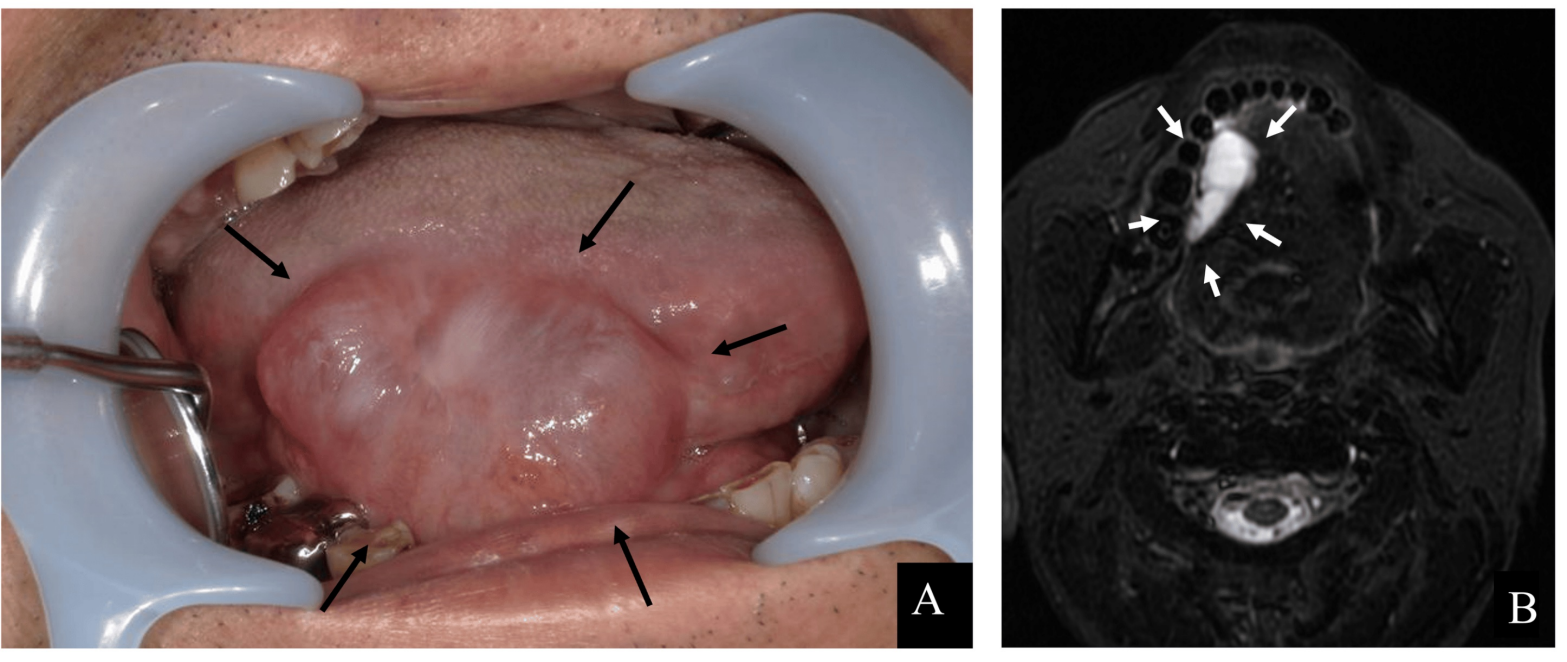
Intraoral photographs and MRI of ranula that appeared seven months after tongue cancer surgery. (A) An intraoral photograph was taken eight months after the initial visit (seven months after tongue cancer surgery). A 38 x 26 mm blue-purple mass was seen at the right lingual border. (B) Contrast-enhanced MRI, T2-weighted image was taken eight months after the initial visit (seven months after tongue cancer surgery). A cystic high-signal area suggestive of fluid retention was observed in the right sublingual crevice.

**Figure 4:**
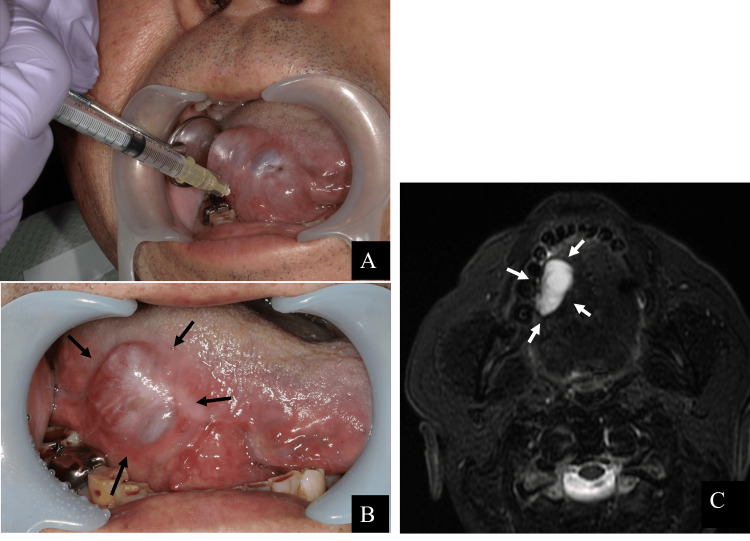
Treatment process of sclerotherapy with OK-432 for intraoral ranula. (A) An intraoral photograph was taken during initial OK-432 sclerotherapy, nine months after the initial visit (eight months after tongue cancer surgery). OK-432 1KE with 0.2 mL saline was injected into the ranula with a 30G needle. (B) Intraoral photograph was taken after the third dose of OK-432, 12 months after the initial visit (11 months after tongue cancer surgery), showing a recurrence of a 20 x 15 mm blue-purple mass. (C) Contrast-enhanced MRI, T2-weighted image was taken after the third dose of OK-432, 12 months after the initial visit. The mass had shrunk slightly but recurred.

The patient’s postoperative course was uneventful. However, seven months after tongue cancer surgery (eight months after the initial visit), the patient noticed a well-defined, soft, blue-purple mass measuring 38 × 26 mm, with an enlarging and shrinking border extending from the right lingual border to the sublingual area (Figure 3A). Therefore, an MRI was performed. The T2-weighted image shows a high-signal cystic area in the right sublingual space, suggesting fluid retention. The interior of the cyst was full; however, there was no wall thickening or enhancement indicating malignancy (Figure 3B). Based on clinical and imaging findings, an intraoral ranula was diagnosed. As the patient initially refused additional surgery at the site of the lesion, a decision was made to perform sclerotherapy. In this therapy, OK-432 is injected into the lesion and is minimally invasive compared with surgery. The first sclerotherapy with OK-432 was performed during the patient's hospitalization, eight months after tongue cancer surgery (nine months after the initial visit). OK-432 1 KE was suspended in 0.2 ml of saline and injected into the cyst cavity using a 30-gauge needle (Figure 4A). No allergic reactions were observed after administration. Although resolution of the ranula was noted after one week following the injection, one month after the initial OK-432 injection, a well-defined, soft, blue-purple mass measuring 25 × 15 mm appeared on the right lingual border. Recurrence was confirmed. As the size of the recurring ranula decreased after the first dose, we determined that the treatment was effective and performed a second OK-432 injection nine months after the tongue cancer surgery (10 months after the initial visit). However, recurrence was observed one month after the second injection, following initial resolution. Nevertheless, we observed a reduction in the maximum diameter of the ranula, from approximately 38 to 20 mm. Therefore, a third OK-432 injection was planned. The third injection was administered 11 months after tongue cancer surgery (12 months after the initial visit). As resolution of the ranula was not observed after three sclerotherapy sessions with OK-432, we concluded that further sclerotherapy was ineffective (Figures 4B, 4C). Therefore, surgery was decided. Owing to complications, the patient preferred to undergo marsupialization rather than sublingual adenectomy. Therefore, marsupialization was planned as the first option.

**Figure 5:**
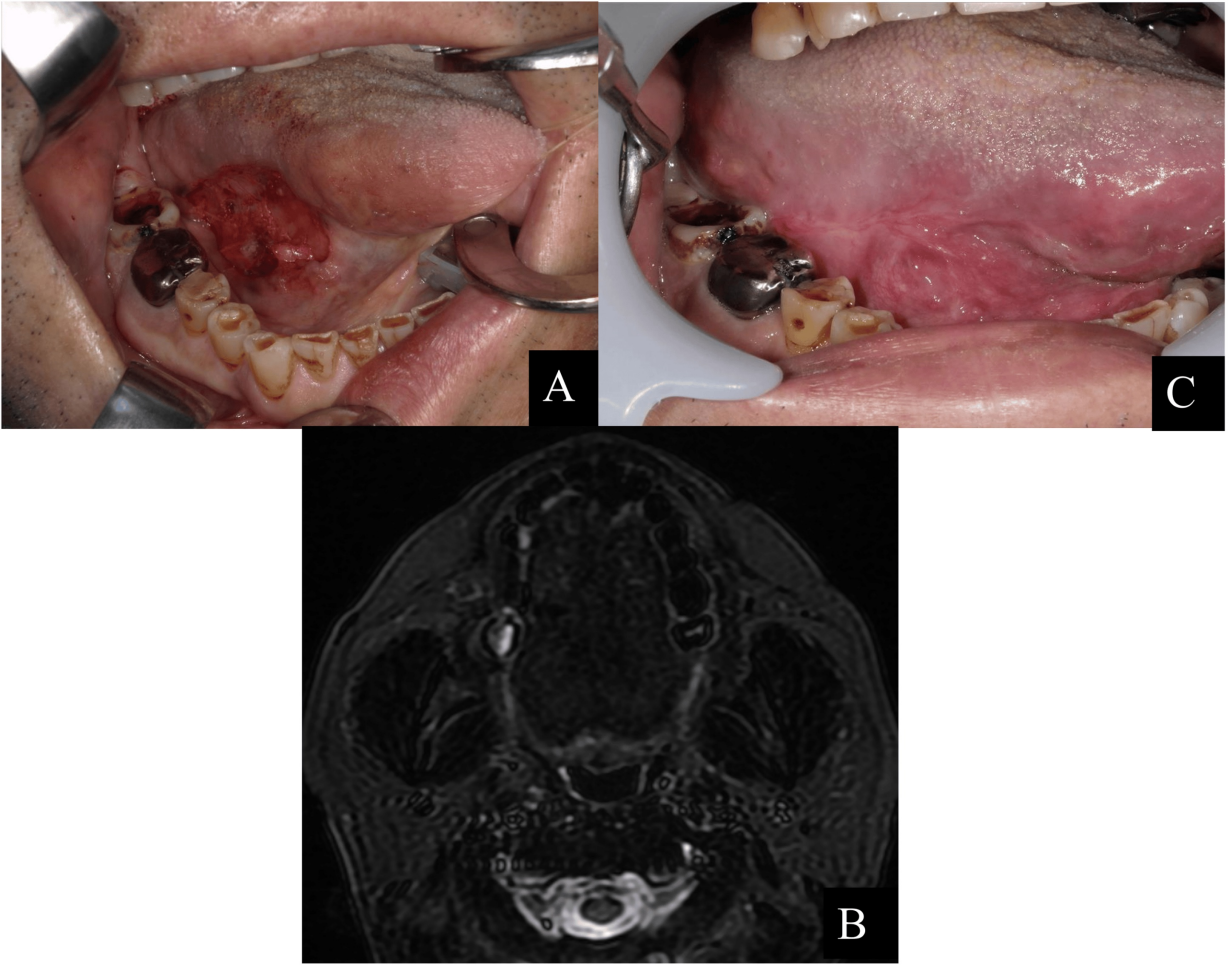
Treatment process of marsupialization for intraoral ranula. (A) An intraoperative intraoral photograph was taken 14 months after the initial visit (12 months after tongue cancer surgery). The ranula marsupialization was performed under general anesthesia. (B) A contrast-enhanced MRI, T2-weighted image was taken 17 months after the initial visit (three months after ranula marsupialization). No recurrence of either ranula or tongue cancer was noted. (C) An intraoral photograph was taken 26 months after the initial visit. No recurrence of either ranula or tongue cancer has been noted to date.

Thirteen months after tongue cancer surgery (14 months after the initial visit), right-sided ranula marsupialization was performed under general anesthesia. Using a No. 11 scalpel, the mucosa corresponding to the cyst canopy was incised while the tongue was pulled forward. The superficial mucosal layer was detached. The mucosa was then detached from the back of the tongue to a depth of approximately 30 mm. Although the lingual nerve and Walton's duct were not exposed, some sublingual glands were present. Using mosquito forceps, the exposed sublingual glands were detached from the surrounding tissues and removed (Figure 5A). The wound was covered with gauze coated with terramycin ointment, and the surgery was completed. One week after surgery, the gauze was removed and the wound was epithelialized. Contrast-enhanced MRI was performed three months after marsupialization (17 months after the initial visit) to confirm the absence of recurrence of the ranula, tongue cancer, and metastasis (Figure 5B). It has now been 12 months since the marsupialization, and there has been no recurrence of ranula or tongue cancer (Figure 5C).

## Discussion

A ranula is a cystic swelling caused by damage to the duct of the sublingual gland and leakage of saliva from the ducts and glands [1]. Treatment modalities for managing intraoral ranula can be divided into two categories: surgical treatment and sclerotherapy with OK-432. Surgical treatments include marsupialization, cystectomy, and sublingual adenectomy [4]. Marsupialization and cystectomy are simple procedures with a low risk of injury to Walton's duct and lingual nerve. However, sublingual adenectomy is a more extensive procedure than marsupialization or cystectomy. In addition, the possibility of complications, such as Walton's duct injury and dysgeusia due to lingual nerve injury, is higher than that of marsupialization or cystectomy, with 11% for lingual nerve paresthesia and 2.8% for Walton's duct injury [11]. However, in terms of cure rate, sublingual adenectomy (98.84%, 95% CI: 96.74 - 99.98%, I^2 ^= 0%) is better than marsupialization or cystectomy (93.85%, 95% CI: 84.18 - 99.15%, I^2^ = 45.97%) [4]. Patel et al. [12] also reported the recurrence rates of each treatment modality for ranula, showing the following rates: marsupialization, 19.8% (n = 86); cystectomy, 20% (n = 5); sublingual adenectomy, 0% (n = 146); and OK-432 sclerotherapy, 57.5% (n = 40). In this case, OK-432 was initially applied, but was ineffective; thus, marsupialization was indicated. Although sublingual adenectomy has a better recurrence rate, marsupialization was performed first in this case because of complications.

OK-432 was approved in Japan in 1975 as an immunotherapeutic agent for cancer [5]. It is currently used to extend the lives of patients with gastrointestinal and lung cancers [13]. It is also administered via intratumoral injections in patients with head and neck or thyroid cancers who fail to respond to other drugs [14,15]. OK-432 causes severe inflammation at the injection site, which is believed to obstruct salivary leakage from the sublingual gland and inhibit salivary influx into the ranula [16]. In the present case, the patient underwent surgery for tongue cancer. In addition to the expected spontaneous resolution of the ranula, OK-432 was prescribed to strengthen antitumor immunity and prevent recurrence [17].

Fukase [16] reported a case in which sclerotherapy with OK-432 was ineffective against recurrent ranulae following surgery. This report suggests that scar formation from surgery renders the surrounding tissue fragile, causing the area to rupture and the drug to leak, resulting in an unsuccessful attempt to close the salivary leak. Kono et al. [18] reported that the ranula length was 17.38 ± 7.10 mm in the OK-432 response group and 29.2 ± 11.09 mm in the OK-432 failure group. This finding indicates that a larger ranula is associated with less successful OK-432 sclerotherapy and a higher recurrence rate. Since the ranula is a pseudocyst that lacks an epithelium, the large ranula may not have been able to withstand the internal pressure caused by the reactive swelling produced by the inflammation induced by OK-432. This patient underwent surgery for tongue cancer. It can be inferred that the sublingual gland conduit was partially damaged owing to the extent of tongue cancer resection and that the perilingual tissue around the ranula was fragile due to postoperative formation of the right-sided, sublingual gland-derived ranula. The long diameter of the ranula was 38 mm, which was larger than the mean ± SD of the OK-432-ineffective group reported by Kono et al. [18]. Thus, it can be considered that, in this case, the fragile ranula was unable to withstand the reactive swelling caused by OK-432 and ruptured before sealing the salivary leak. As a result, the drug leaked out, rendering sclerotherapy ineffective and resulting in recurrence. Therefore, sclerotherapy with OK-432 may be less effective for treating large postoperative ranulae in patients with tongue cancer.

Surgery, particularly sublingual adenectomy, is considered the best treatment option in cases demonstrating recurrence after multiple treatments with OK-432. Surgery is practically considered the most reliable treatment method as it eliminates the source of saliva leakage. Chung et al. [4] and Patel et al. [12] reported that sublingual adenectomy is an effective treatment method with low recurrence rates. However, Zhao et al. [11] reported that sublingual adenectomy is associated with a higher probability of salivary fistula due to Walton's duct injury and dysesthesia or dysgeusia due to lingual nerve injury, compared to other surgical procedures for ranula. Therefore, a thorough consultation emphasizing the potential benefits and risks of each treatment method should be conducted with the patients before selecting an optimal treatment plan.

The limitation of this study is that, due to the small number of cases (only one) and the absence of long-term follow-up, it is not possible to conclude with certainty that OK-432 is ineffective in patients following tongue cancer surgery and that surgical treatment should be recommended. To the best of our knowledge, no study has evaluated the efficacy of OK-432 for ranulae that develop after tongue cancer surgery. Therefore, additional follow-up and case studies are warranted. However, based on the report by Fukase [16] indicating the ineffectiveness of OK-432 in cases of recurrent ranula following surgical treatment, it is anticipated that OK-432 may also be ineffective for ranulae occurring in the same surgical field after resection or other oral mucosal surgeries, and that surgical treatment may be the preferred option. Therefore, we believe that it would be useful to compare the effectiveness of OK-432 and surgical treatment for managing ranulae occurring in the same surgical field following oral mucosal surgery, such as partial resection of tongue cancer.

## Conclusions

We encountered a case of refractory large ranula that developed after surgery for tongue cancer. Sclerotherapy with OK-432 has recently been used to treat ranula because it is less invasive than surgical treatments. In similar postoperative cases where the ranula is large or tissue integrity is compromised, surgical treatment may be more effective than sclerotherapy. However, since this is only a single case report and with no long-term follow-up, further research with a longer follow-up duration is warranted on the treatment methods for similar cases.
